# Wnt Glycation Inhibits Canonical Signaling

**DOI:** 10.3390/cells8111320

**Published:** 2019-10-25

**Authors:** Zhennan Ye, Sonnhild Mittag, Martin Schmidt, Andreas Simm, Rüdiger Horstkorte, Otmar Huber

**Affiliations:** 1 Department of Biochemistry II, Jena University Hospital, Friedrich Schiller University Jena, 07743 Jena, Germany; zhennan.ye@med.uni-jena.de (Z.Y.); sonnhild.mittag@med.uni-jena.de (S.M.); martin.schmidt@med.uni-jena.de (M.S.); 2 Department of Cardiac Surgery, Middle German Heart Center, University Hospital Halle, Martin Luther University Halle-Wittenberg, 06120 Halle/Saale, Germany; andreas.simm@uk-halle.de; 3 Institute for Physiological Chemistry, Martin Luther University Halle-Wittenberg, 06114 Halle/Saale, Germany; ruediger.horstkorte@medizin.uni-halle.de

**Keywords:** Wnt3a, β-catenin, glyoxal, methylglyoxal, glycation, advanced glycation end products, AGEs, RAGE, aging

## Abstract

Glycation occurs as a non-enzymatic reaction between amino and thiol groups of proteins, lipids, and nucleotides with reducing sugars or α-dicarbonyl metabolites. The chemical reaction underlying is the Maillard reaction leading to the formation of a heterogeneous group of compounds named advanced glycation end products (AGEs). Deleterious effects have been observed to accompany glycation such as alterations of protein structure and function resulting in crosslinking and accumulation of insoluble protein aggregates. A substantial body of evidence associates glycation with aging. Wnt signaling plays a fundamental role in stem cell biology as well as in regeneration and repair mechanisms. Emerging evidence implicates that changes in Wnt/β-catenin pathway activity contribute to the aging process. Here, we investigated the effect of glycation of Wnt3a on its signaling activity. Methods: Glycation was induced by treatment of Wnt3a-conditioned medium (CM) with glyoxal (GO). Effects on Wnt3a signaling activity were analyzed by Topflash/Fopflash reporter gene assay, co-immunoprecipitation, and quantitative RT-PCR. Results: Our data show that GO-treatment results in glycation of Wnt3a. Glycated Wnt3a suppresses β-catenin transcriptional activity in reporter gene assays, reduced binding of β-catenin to T-cell factor 4 (TCF-4) and extenuated transcription of Wnt/β-catenin target genes. Conclusions: GO-induced glycation impairs Wnt3a signaling function.

## 1. Introduction

Aging-associated disabilities are generated by an accumulation of randomly occurring molecular damages during life time, which affect the organism at multiple levels such as metabolism, immune response, repair, and regeneration as well as altered inter- and intracellular communication. Typical alterations detectable in aged tissues are increased concentrations of reactive oxygen species (ROS) and concomitant increased DNA-damage, lipid and protein oxidation [1,2]. Often this is accompanied by accumulation of insoluble protein aggregates (lipofuscin) [3,4] at least partially as a consequence of impaired proteasome function [5]. In addition, glycation was reported to contribute to limited degradation and function of proteins [6]. Glycation represents an important posttranslational modification that significantly increases during aging and results in the formation of advanced glycation end products (AGEs).

AGEs are generated by an initial non-enzymatic reaction of amino groups with reactive carbonyl groups of monosaccharides known as the Maillard reaction [7], thereby forming Schiff-base adducts. These adducts are subsequently rearranged to so-called Amadori products which undergo further oxidation and dehydration reactions leading to a large group of heterogeneous products. Up to the stage of the Amadori products, adducts are basically reversible. Preferentially lysine residues, but also other amino acids including arginine, histidine, tryptophan, or cysteine, are modified by glycation [8]. The most prominent and well-known AGEs identified in cells and tissues are carboxymethyllysine (CML), carboxyethyllysine (CEL), pentosidine, and desoxyglucosone. Physiologically, these AGEs are formed in response to so-called “dicarbonyl stress” [9] as given under conditions of hyperglycemia or in the presence of highly reactive α-dicarbonyls (α-DCs) such as glyoxal (GO), methylglyoxal (MGO) or 3-deoxyglucosone [10]. These α-DCs are inevitable byproducts of anaerobic glycolysis and lipid peroxidation and in consequence, induce the formation of highly stable AGEs [11] which crosslink proteins and lead to protease-resistant protein aggregates [12,13]. In this context, glycation-induced modifications of extracellular matrix proteins resulting in intermolecular crosslinks lead to increased stiffness of tissues and vessels and affect cell migration [14]. Furthermore, there is clear evidence that glycation and AGEs lead to mitochondrial dysfunction [15,16] and impaired immune responses [17,18,19]. Glycation of growth factors such as platelet-derived growth factor (PDGF) [20] and insulin [21,22,23], as well as receptors such as nerve growth factor (NGF)-receptor [24], were reported to affect signaling functions. 

In addition, AGE modification itself can induce signaling mainly through the receptor for advanced glycation end products (RAGE), a multi-ligand transmembrane receptor containing immunoglobulin domains [11,25]. RAGE-induced signaling contributes to aging-related inflammation and metabolic diseases by modulation of multiple pathways including phosphoinositide 3-kinase (PI3K), Erk1/2 [26,27] and Wnt signaling [28,29]. However, the outcome of AGEs and RAGE signaling appears to be a double-edged sword with degenerative and protective effects [9,30]. This also appears to be true in respect to the role of Wnt signaling in aging [31].

The canonical Wnt/β-catenin signaling cascade acts as a pivotal regulator of developmental and pathogenesis pathways including cell stemness maintenance/differentiation, carcinogenesis, and aging-related phenotypes [32,33]. Moreover, there is clear evidence for a role of the Wnt pathway in mitochondrial respiratory capacity [34] and metabolic homeostasis, especially of glucose metabolism [35]. Within the canonical Wnt signaling pathway, β-catenin is the central player. Normally, excess β-catenin is rapidly degraded by the ubiquitin-proteasome pathway [36]. Activation of the pathway leads to the stabilization of β-catenin and its accumulation within the cytosol and the nucleus, where it interacts with T-cell factor/lymphoid enhancer-binding factor (TCF/LEF) transcription factors [37,38,39] and thereby regulates transcription of multiple target genes. During recent years, a multitude of other crosstalks and co-operations affecting the pathway have been identified [40]. Interestingly, variants of the TCF-4 (TCF7L2) gene have been associated with increased risk of type 2 diabetes [41].

Based on that knowledge, we hypothesized that Wnt ligands as secreted, cysteine-rich proteins [42] are susceptible to α-DC-mediated modifications and that glycation might modulate their signaling activities. For this study, we decided to use Wnt3a as a proof of principle to study effects of GO-treatment on Wnt3a conditioned medium (Wnt3a CM). We observed that the ratio of phospho-β-catenin to total β-catenin increased. As a further consequence, Wnt3a-induced activation of the β-catenin/TCF transcription complex was reduced as shown by reporter gene assays and by decreased expression of endogenous Wnt target genes.

## 2. Materials and Methods

### 2.1. Cell Culture

Human embryonic kidney (HEK)-293 and Neuro-2a cells were maintained in high glucose Dulbecco’s Modified Eagle Medium (DMEM) (Sigma-Aldrich, Steinheim, Germany) supplemented with 10% (v/v) fetal bovine serum (FBS) (PAN-Biotech, Aidenbach, Germany) and 100 U/ml penicillin, 100 mg/ml streptomycin (Sigma) in a humidified 5% CO_2_ atmosphere at 37 °C.

### 2.2. Preparation of Wnt3a-Conditioned Medium (Wnt3a CM)

Wnt3a-secreting cells (L-M(TK-)Wnt3A) and the corresponding empty vector control cell line were cultured in high glucose DMEM as mentioned above to produce Wnt3a CM and control CM [42]. Briefly, cells were seeded in 10 cm dishes at a 1:10 split. After 4 days of culture, the first batch of CM was harvested. Cells were further incubated with replenished medium for additional 3 days. After this, the second batch of CM was collected. CM was centrifuged at 2.500× *g* for 10 min. The abundance of the Wnt3a protein in CM was confirmed by Western blotting and luciferase reporter gene assays. The conditioned medium was stored at 4 °C for further experiments. Only the second batch of conditioned medium was applied in the experiments. Before GO-treatment, CM was diluted 1:2 with fresh complete DMEM.

### 2.3. GO-Treatment of Wnt3a CM and Recombinant Human Wnt3a

Glyoxal (GO) and methylglyoxal (MGO) were purchased from Sigma-Aldrich (Steinheim, Germany). For glycation, conditioned media were pre-incubated with the indicated concentrations of GO or MGO at 37 °C for 3 h and subsequently added to either HEK-293 or Neuro-2a cells for another 18 h. In a second approach, medium was first incubated with 1 mM or 2 mM GO at 37 °C for 5 h. Then GO was removed by dialysis using membranes with a molecular weight cutoff of 4 kDa (Carl Roth GmbH, Karlsruhe, Germany). Dialysis was conducted overnight in 2 liters of dialysis buffer (phosphate buffered saline (PBS)) at 4 °C. For glycation of purified recombinant human Wnt3a protein (#5036-WN/CF; R&D Systems, distributed by Bio-Techne GmbH, Wiesbaden-Nordenstadt, Germany), 0.5 μg protein was incubated with 2 mM GO for 5 h at 37 °C. Monoclonal anti-carboxymethyllysine (anti-CML26) antibody was obtained from Acris/OriGene Technologies GmbH (Herford, Germany).

### 2.4. Luciferase Reporter Gene Assays

Assays were performed as previously reported [43]. In detail, cells were seeded into a 24 well plate with a density of 1 × 10^5^ cells/well. The next day, cells were co-transfected with 500 ng Topflash/Fopflash (pGL3-OT/OF) luciferase constructs (provided by Dr. Bert Vogelstein, Johns Hopkins University, Baltimore, USA) [44] and 50 ng Renilla luciferase (Promega GmbH, Mannheim, Germany) control plasmid using polyethyleneimine (PEI). Eighteen hours after transfection, cells were treated with glycated Wnt3a CM or glycated control CM as indicated for 24 h. Subsequently, cells were lysed in 150 μl/well passive phenylbenzothiazole (PPBT) buffer (100 mM K_3_PO_4_ pH 7.8, 0.2 % (v/v) Triton X-100) and 20 μl of each sample lysate was separately subjected to Firefly- and Renilla-luciferase measurement using a Mithras LB 940 Multimode Microplate Reader (Berthold Technologies GmbH, Bad Wildbad, Germany).

### 2.5. Immunoprecipitation and Western Blotting

Protein A-sepharose CL-4B (GE Healthcare, Freiburg, Germany) beads were pre-coupled with anti-FLAG M2 antibody (Sigma-Aldrich, Schnelldorf, Germany) overnight at 4 °C. Cells were lysed in immunoprecipitation IP buffer (50 mM Tris/HCl pH 8.0, 150 mM NaCl, 5 mM ethylenediaminetetraacetic acid (EDTA), 1% (v/v) Triton X-100) with freshly added protease (Roche cOmplete, distributed by Sigma-Aldrich, Germany) and phosphatase (Roche PhosSTOP distributed by Sigma-Aldrich, Germany) inhibitors and lysates were then subjected to immunoprecipitation with anti-FLAG M2 pre-conjugated beads for additional 2 h. After 3× washing, precipitated proteins were eluted with 2× sodium dodecyl sulfate (SDS) sample buffer and separated by SDS-polyacrylamide gel electrophoresis (SDS-PAGE) and analyzed by Western blotting with the indicated antibodies. 

To investigate the effects of dialysis on GO-treated conditioned media HEK-293 and Neuro-2a cell lysates were generated after 12 h incubation with dialyzed GO-treated Wnt3a CM and control CM. Cells were solubilized in radioimmunoprecipitation assay (RIPA) buffer (50 mM Tris-HCl pH 7.5, 150 mM NaCl, 0.5% (w/v) sodium deoxycholate, 0.1% (w/v) SDS, 1% (v/v) NP-40) containing protease inhibitor and phosphatase inhibitor cocktails (see above). Western blots were performed using the indicated antibodies anti-β-catenin (C14) (BD BioSciences, Heidelberg, Germany), anti-phospho-β-catenin (Ser33/37) (Cell Signaling Technologies, Frankfurt/Main, Germany), anti- glyceraldehyde 3-phosphate dehydrogenase (GAPDH) (Merck-Millipore, Darmstadt, Germany) according to the manufacturers’ recommendations. Anti-Wnt3a antibody was obtained from R&D Systems (distributed by Bio-Techne GmbH, Wiesbaden-Nordenstadt, Germany).

### 2.6. RNA Isolation and Quantitative Reverse Transcription PCR (RT-PCR)

Total RNA extraction and cDNA synthesis were conducted according to the manufacturer’s instructions using the NucleoSpin RNA II kit (Macherey and Nagel, Düren, Germany) and the High-Capacity cDNA Reverse Transcription Kit (Life Technologies, Darmstadt, Germany). Real-time PCR was performed using Applied Biosystems StepOnePlus system with BRYT Green dye (2-Step RT-qPCR GoTaq® qPCR and RT-qPCR Systems, Promega GmbH, Mannheim, Germany) as described previously [45]. Each PCR was set up in triplicates and performed from at least three independent RNA preparations. Threshold cycle (C_t_) values of the target genes were normalized to glyceraldehyde-3-phosphate dehydrogenase (GAPDH). Differential expression was calculated according to the 2^−ΔΔCt^ method. The following primer pairs were used for the detection of canonical Wnt target genes: *sp5* fwd: 5’-GGGGAGACTCAGCAGACG-3’, rev: 5’-TGGGTCCCTATGTCCGAA G-3’; *axin2* fwd: 5’-CAAGCCTGGCTCCAGAAG-3’, rev: 5’-GCATCCTCCGGTATGGAAT-3’; *cyclin D1* fwd: 5’-GCTGTGCATCTACACCGACA-3’, rev: 5’-TTGAGCTTGTTCACCAGGAG-3’; *GAPDH* fwd: 5’-AGCCACATCGCTCAGACAC-3’, rev: 5’-GCCCAATACGACCAAATCC-3’.

### 2.7. Statistical Analysis

Statistical analyses were performed using SigmaPlot software (Systat Software GmbH, Erkrath, Germany) with results presented as the mean ± standard deviation (SD). P-values of <0.05 were considered statistically significant.

## 3. Results

### 3.1. Glyoxal Treatment Leads to Glycation of Medium Components

**Figure 1:**
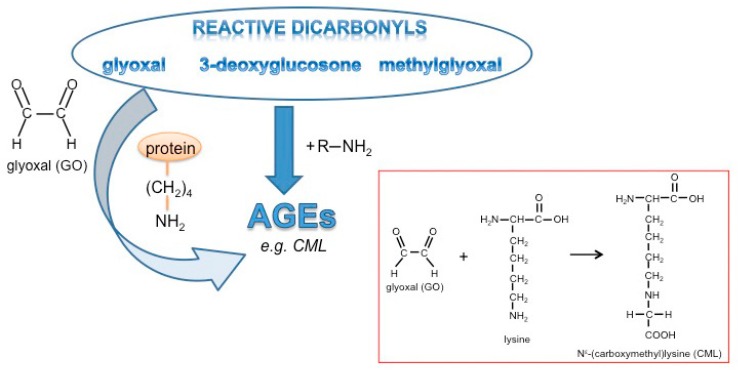
Glycation and formation of advanced glycation end products (AGEs).

**Figure 2:**
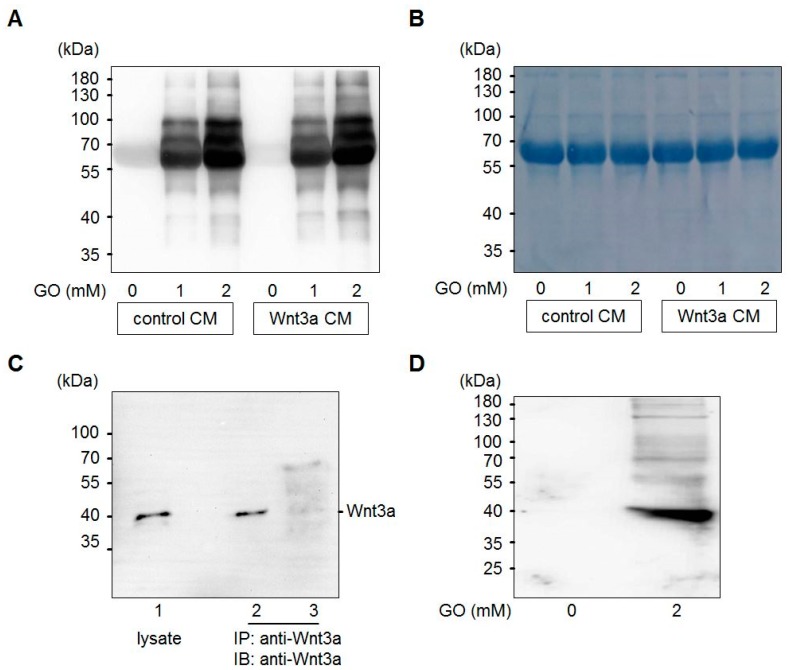
Glycation of conditioned media (CM) with GO. (**A**) Control CM and Wnt3a CM were treated with different concentrations (0, 1, 2 mM) of GO for 5 h and analyzed by SDS-PAGE and Western blotting with anti-CML26 antibody. (**B**) After Western blot detection the same membrane was stained with Coomassie brilliant blue to show equal loading. The strong band corresponds to bovine serum albumin of the cell culture medium. (**C**) Western blot detection of Wnt3a from L-M(TK-)Wnt3A cell lysates (lane 1) or immunoprecipitated from serum-free Wnt3a CM (lane 2) or GO-treated Wnt3a CM (lane 3). (**D**) Treatment of 0.5 μg of purified Wnt3a protein with 2 mM GO induced Wnt3a carboxymethylation as detected with the anti-CML26 antibody on a Western blot. The untreated control was not modified.

Glyoxal is formed in large amounts by lipid peroxidation [46] or at lower levels by slow pH-dependent degradation of glucose [47] and actively reacts with amino-groups of proteins to form advanced glycation end products (AGEs) (Figure 1). Wnt3a is a member of the Wnt family of glycoproteins that are secreted into the cell culture supernatant. This so-called Wnt3a conditioned medium (Wnt3a CM) can be used to stimulate the canonical Wnt signaling pathway in target cells. In a first set of experiments, we tested the consequences of GO-treatment on proteins in Wnt3a CM and control CM in respect to protein glycation. Media were treated with increasing concentrations of GO (0, 1, 2 mM) for 5 h at 37 °C to induce AGE formation. Subsequently, medium components were separated by SDS-PAGE and analyzed by Western blotting with anti-carboxymethyllysine (CML26) antibody to detect at least a fraction of potential AGEs that can form in response to incubation with GO. As shown in Figure 2A, GO-treatment resulted in a dose-dependent increase of glycated proteins of different molecular weights in the conditioned media. After Western blot detection, membranes were stained with Coomassie blue to confirm equal loading (Figure 2B). The major detectable band most likely represents bovine serum albumin. To examine Wnt3a after GO-treatment, Wnt3a was immunoprecipitated from serum-free GO-treated Wnt3a CM and analyzed by Western blotting with anti-Wnt3a antibody. GO-treatment resulted in a ladder of bands with a shift to higher molecular masses (Figure 2C). The amount of precipitated Wnt3a appears to be lower than the protein precipitated from supernatants not preincubated with GO. This is probably due to less efficient binding of modified Wnt3a to the anti-Wnt3a antibody or impaired solubility. When we analyzed immunoprecipitated Wnt3a from GO-treated CM with anti-CML26 antibody, we could not detect a clear glycation signal. However, it was possible to show that Wnt3a can be directly modified by this non-enzymatic reaction when we treated partially purified Wnt3a (bovine serum albumin free (BSA)-free) with GO and directly analyzed the protein with the anti-CML26 antibody on a Western blot. A strong signal was detectable for GO-treated Wnt3a. In contrast, when the same amount of untreated Wnt3a protein was loaded as a control, no signal was detectable (Figure 2D).

### 3.2. GO-Treatment Inhibits the Activity of Wnt3a-Conditioned Medium

**Figure 3:**
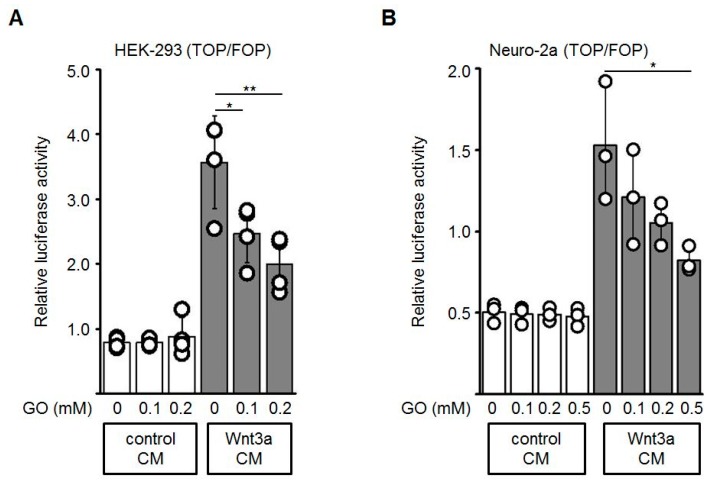
The stimulating activity of Wnt3a-conditioned media (Wnt3a CM) was suppressed after GO-treatment in reporter gene assays. HEK-293 (**A**) or Neuro-2a (**B**) cells were co-transfected with Topflash (TOP) or Fopflash (FOP) plasmids together with the Renilla plasmid, which was used as an internal control for transfection efficiency. Prior to control CM and Wnt3a CM stimulation, media were pre-incubated with indicated concentrations of GO for 3 h at 37 °C, then added to the transfected cells for additional 18 h. Luciferase reporter activity was measured. The graphs show mean values ±SD of n = 3 independent experiments, each measured in duplicate. Circles represent individual experiments. Differences to non-glycated Wnt3a CM were analyzed by one-way ANOVA analysis. * *p* < 0.05, ** *p* < 0.01.

To test if the activity of Wnt3a is altered by GO-treatment, Topflash/Fopflash (TOP/FOP) reporter gene assays in HEK-293 and Neuro-2a cells were performed. The Topflash reporter plasmid contains three TCF/LEF response elements whereas in the Fopflash plasmid these sites are mutated. Activation of the Wnt/β-catenin pathway induces binding of β-catenin to TCF/LEF and thereby activates transcription of the Firefly luciferase reporter gene. First, HEK-293 or Neuro-2a cells were transiently co-transfected with Topflash or Fopflash and Renilla luciferase (for normalization of transfection efficiency) plasmids. Wnt3a CM and control CM were pre-incubated with different concentrations of GO (0, 0.1, 0.2, 0.5 mM) for 3 h. The GO-treated media were subsequently diluted 1:2 with fresh medium and added to the reporter plasmid-transfected cells. GO-treatment of Wnt3a CM induced a dose-dependent reduction of the Wnt3a-stimulated transcriptional activity, both in HEK-293 (Figure 3A) and Neuro-2a (Figure 3B) cells, while the control CM did not show major changes in response to GO-treatment. Different batches of Wnt3a CM revealed similar results. Similar experiments were performed with increasing concentrations of MGO and again revealed a dose-dependent reduction of the reporter gene activity (Appendix A).

**Figure 4:**
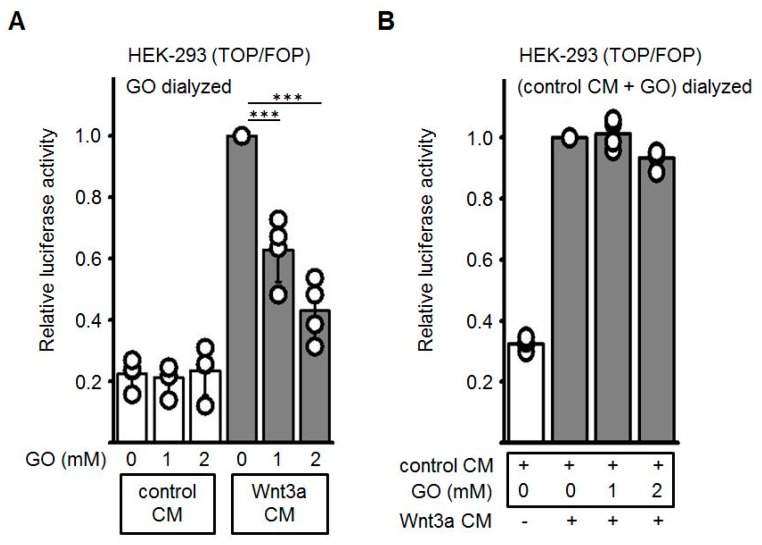
Effect of dialysis on GO-treated CM. Conditioned media were incubated with GO for 5 h at 37 °C and subsequently dialyzed overnight against PBS at 4 °C. (**A**) After GO removal, dose-dependent reduction of glycated Wnt3a CM activity was still detectable in Topflash/Fopflash reporter gene assays. (**B**) Dialyzed GO-treated control CM did not inhibit luciferase activity induced by non-glycated Wnt3a CM. Values of non-glycated Wnt3a CM were normalized to 1. The graphs show mean values ±SD of n = 4 independent experiments, each measured in duplicate. Circles represent individual experiments. Differences to non-glycated Wnt3a CM were analyzed by paired t-test. *** *p* < 0.001.

Next, we wanted to exclude that the GO, which is still present in the Wnt3a CM, may modify the Frizzled/ low-density lipoprotein-related protein (LRP) receptor complex at the cell surface and thereby block receptor-mediated signal transduction. Therefore, GO-pretreated cell culture media were subjected to dialysis to remove excess GO. As shown in Figure 4A, after removal of GO the dose-dependent repressive effect of GO-pretreated Wnt3a CM was comparably to non-dialyzed CM (see Figure 4A). Finally, glycated CM after dialysis was combined with non-glycated Wnt3a CM. In this combination the activating property of Wnt3a CM was not impaired (Figure 4B). Again, comparable results were obtained when different batches of Wnt3a CM were used. These observations suggested that glycation of the Wnt3a protein accounts for the inhibitory effect whereas neither GO per se nor other AGEs generated by GO-treatment of control CM contribute to the inhibitory effect.

### 3.3. Glycation of Wnt3a CM Affects Levels of Total β-Catenin and Phospho-β-Catenin

**Figure 5:**
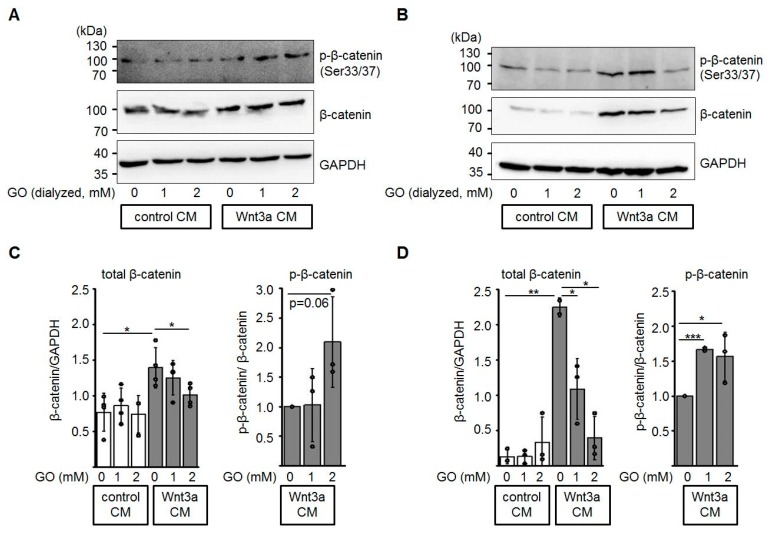
Glycation of Wnt3a CM affects total β-catenin and phospho-β-catenin levels. HEK-293 (**A**, **C**) and Neuro-2a (**B**, **D**) cells were treated with dialyzed conditioned medium for 12 h and cell lysates were subjected to SDS-PAGE and Western blotting as indicated. The presented blots are representatives of n ≥ 3 independent experiments. Circles represent individual experiments. * *p* < 0.05, ** *p* < 0.01, *** *p* < 0.001.

In the absence of a Wnt ligand, excess β-catenin is phosphorylated by casein kinase 1 (CK1) and glycogen synthase kinase-3β (GSK3β) within the β-catenin destruction complex and subsequently degraded by the ubiquitin-proteasome pathway [36,48]. In this context, we investigated the protein levels of total β-catenin and phosphorylated β-catenin in HEK-293 and Neuro-2a cells after incubation with GO-treated media (Figure 5). In the presence of glycated Wnt3a CM, total β-catenin protein levels were reduced in a dose-dependent manner, while the amount of phospho-β-catenin (Ser33/Ser37) relative to total β-catenin increased.

### 3.4. GO-Treated Wnt3a CM Affects Binding of β-Catenin to TCF-4

**Figure 6:**
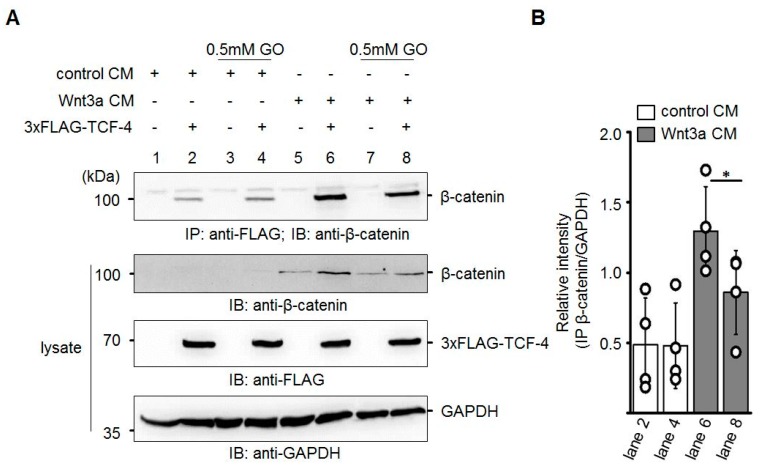
Reduced binding of β-catenin to TCF-4 after GO-treatment of Wnt3a CM. (**A**) Neuro-2a cells were transiently transfected with 3xFLAG-TCF-4 and subsequently treated with the indicated CM. After immunoprecipitation (IP) with anti-FLAG antibody, the amount of co-precipitating β-catenin was analyzed by immunoblotting (IB). The presented blot is a representative of n = 4 independent experiments. (**B**) Quantification of Western blot experiments. Signal of co-immunoprecipitated β-catenin was normalized to GAPDH in the lysates. The graph shows mean values ±SD. Circles represent individual experiments. Data were analyzed with a paired t-test. * *p* < 0.05.

In line with the inhibition of transcriptional activity and the reduced amount of total β-catenin detected in response to GO-treatment of Wnt3a CM, we assumed that β-catenin-binding to TCF-4 was attenuated. To test this, Neuro-2a cells were transiently transfected with N-terminal 3xFLAG-tagged TCF-4 and harvested 24 h later. TCF-4 was immunoprecipitated with Protein A-sepharose-CL-4B bound anti-FLAG M2 antibody and analyzed for co-precipitated endogenous β-catenin by Western blotting. Application of GO (0.5 mM)-treated Wnt3a CM resulted in the reduced co-immunoprecipitation of β-catenin (Figure 6).

### 3.5. Reduced Expression of Wnt Target Genes by GO-Treated Wnt3a CM

**Figure 7:**
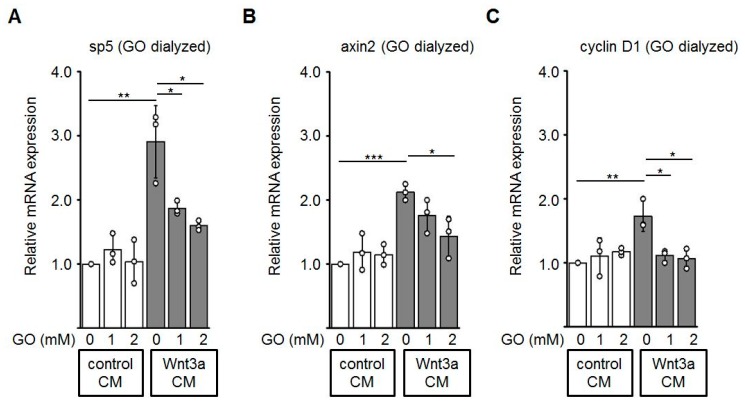
Expression of the endogenous Wnt target genes *sp5* (**A**), *axin2* (**B**), and *cyclin D1* (**C**) is down-regulated in HEK-293 cells incubated with glycated Wnt3a CM (GO dialyzed). The graphs show mean values ±SD. Circles represent individual experiments. n ≥ 3, * *p* < 0.05, ** *p* < 0.01.

Finally, we performed quantitative RT-PCR to evaluate effects of glycated Wnt3a CM on the mRNA expression of known Wnt/β-catenin target genes including *sp5* [49], *axin2* [50], and *cyclin D1* [51] in HEK-293 cells. Consistent with the assays described above, Wnt3a CM up-regulates expression of the analyzed Wnt target genes. Glycation of Wnt3a CM with GO alleviated the expression of these target genes in a dose-dependent manner (Figure 7).

## 4. Discussion

Protein glycation is a spontaneous, non-enzymatic reaction of reducing sugars or α-DCs with preferentially lysine, cysteine, and arginine residues in proteins, finally resulting in a set of different modifications called AGEs. In consequence, protein structure and function are altered and often lead to the accumulation of damaged macromolecules [52]. This study aims to determine whether Wnt3a as a representative canonical Wnt family member can be modified by glycation and how this affects its signaling activity. Wnt signaling regulates multiple processes crucial for embryogenesis, tissue homeostasis and repair. Modulation of Wnt signaling may contribute to aging-associated impairment of repair and regeneration and exhaustion of stem cells [31]. In this context, it has been shown that intestinal stem cell regenerative capacity is reduced upon aging because of reduced canonical Wnt signaling [53].

In this study, in vitro glycation of Wnt3a in cell culture supernatants isolated from Wnt3a-secreting L-M(TK-) cells [42] was induced by addition of GO. Since this supernatant contained serum components of the cell culture medium, supernatants of L-M(TK-) cells not overexpressing Wnt3a were used as control CM to exclude that AGEs formed in response to GO-treatment are responsible for the detectable effects. Untreated Wnt3a CM induced luciferase activity in reporter gene assay as reported previously [45,54]. GO-treatment however, diminished the capacity of the Wnt3a CM to activate transcription. A similar effect was observed for MGO. Glycation of medium components obviously was not involved in the impairment as glycated control CM did not repress Wnt3a CM-induced transcription. In addition, it can be excluded that AGEs generated in the glycated control cell culture supernatant activated RAGE and, in this way, induced the observed repressive effect. Moreover, when GO-treated Wnt3a CM was dialyzed to remove the excess GO before addition to the reporter cells, again a repressive effect was detectable. Glycation of components on the cell surface or within the cells thus appears to not be involved in the observed reduction of the Wnt signaling activity. Unfortunately, we did not succeed in proving that Wnt3a from conditioned media is glycated, even when concentrated by immunoprecipitation. This may be due to the low concentration and solubility of Wnt proteins. However, recombinant Wnt3a itself is glycated in response to GO-treatment, as shown by Western blot analysis with an anti-carboxymethyllysine antibody. Future studies have to show if sufficient amounts of endogenous Wnt proteins can be isolated to detect glycation, for instance by mass spectrometry. In this context, it has to be considered that Wnt itself may represent an AGE, which can bind directly to AGE-receptors (RAGEs). Therefore, it cannot be excluded that RAGE signaling contributes to the inhibitory effect. 

Taken together, our data indicate that glycation of Wnt3a impairs its signaling activity. Consistently, we observed that addition of glycated Wnt3a CM resulted in increased phospho-β-catenin and reduced total β-catenin levels, and consistently a reduced amount of β-catenin binding to TCF-4. This led to a reduced expression of endogenous Wnt target genes as shown for *sp5* (specificity protein 5) [49], *axin2* [50] and *cyclin D1* [51] by quantitative RT-PCR. Cyclin D1 and the transcription factor sp5 are involved in regulation of cell proliferation. Sp5 was reported to act as a context-dependent activator or inhibitor of specific sets of Wnt target genes [55,56]. Axin2 as a component of the β-catenin degradation complex acts as a feedback inhibitor of the canonical Wnt signaling pathway [57].

**Figure 8:**
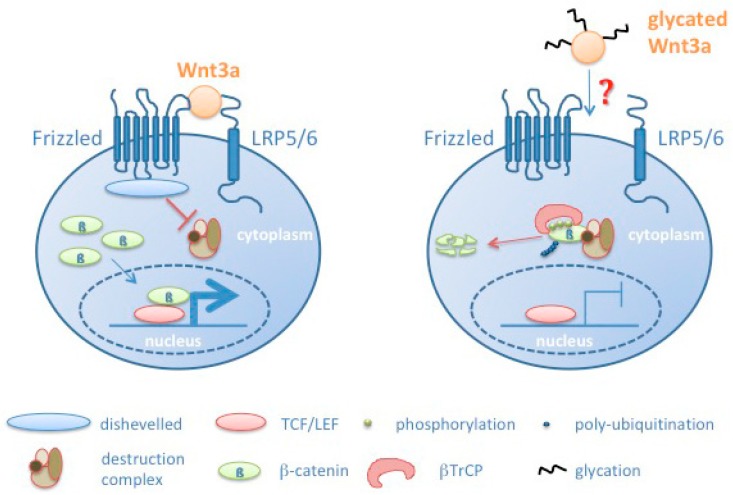
Schematic model summarizing effects induced by Wnt glycation, resulting in increased phosphorylation of β-catenin, its subsequent degradation and reduced transcription of Wnt target genes. βTRCP: β-transducin repeat containing protein.

All our findings correlate with a reduced signaling activity as a consequence of Wnt3a glycation (see model in Figure 8). However, at the moment we cannot explain how glycation of Wnt3a leads to reduced signaling. Different modes have to be considered. Wnts as cysteine-rich proteins are potential targets for oxidative damage and glycation. Oxidation and glycation of Wnt3a may inhibit its binding to the Frizzled/LRP6 receptor complex due to structural changes in the Wnt3a protein. In addition, Wnts are modified by glycosylation and lipid modifications [42]. Specifically, the lipid modifications turn Wnts to hydrophobic molecules. Currently it is not fully understood how Wnts are transported from the producing cell to the target cells [58]. Glycation may affect processes associated with Wnt transport. Moreover, glycation may impair its already low solubility and induce its precipitation. Preliminary assays to address the aspect of solubility in response to glycation however, do not allow to finally confirm this assumption. Currently, it is also unknown how glycation affects the half-life of Wnt molecules.

Future studies have to confirm our in vitro results in vivo to justify that Wnt ligands indeed can effectively be glycated in different processes during aging, such as impaired repair and stem cell regeneration, or may contribute to the pathogenesis of diseases such as diabetes, osteoporosis, or neurological disorders [59]. Nevertheless, this novel finding on Wnt3a glycation and its effect on Wnt signaling sheds light on a new molecular mechanism that alters intercellular communication, a hallmark of aging.

## Figures and Tables

**Figure A1:**
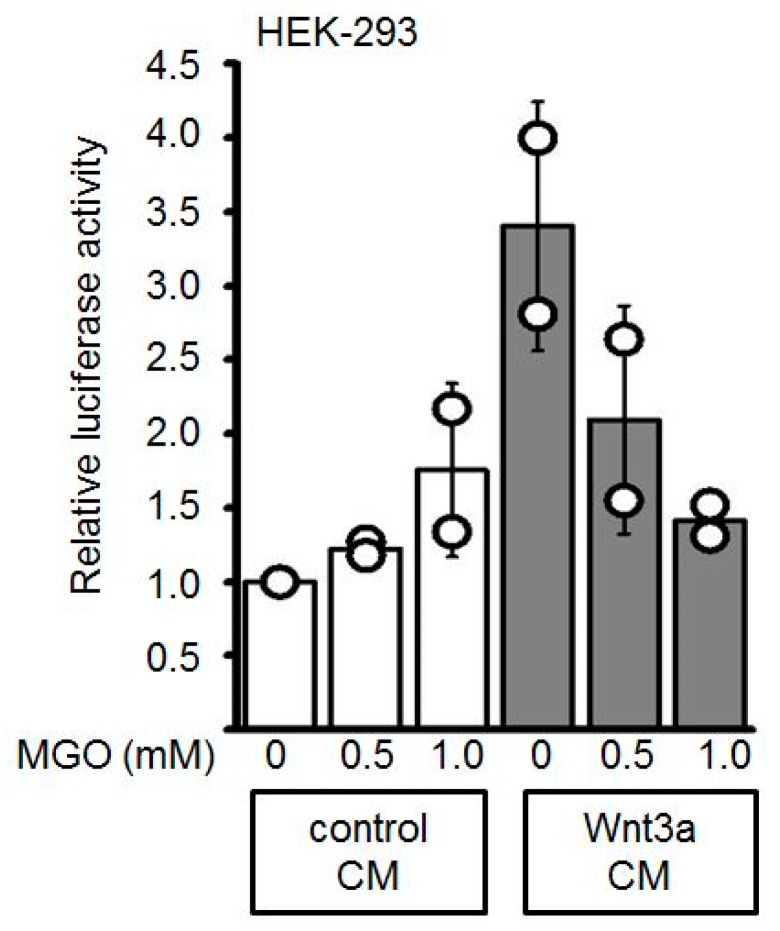
The stimulating activity of Wnt3a-conditioned media (Wnt3a CM) was suppressed after MGO-treatment in reporter gene assays. The activity of Wnt3a-conditioned media (Wnt3a CM) was suppressed after methylglyoxal (MGO)-treatment in Topflash/Fopflash reporter gene assays in HEK-293 cells. The graph shows mean values ±SD. Circles represent individual experiments. n = 2.

## Abbreviations

α-DCs	α-dicarbonyls
AGE	advanced glycation end product
βTRCP	β-transducin repeat containing protein
BSA	bovine serum albumin
CEL	carboxyethyllysine
CK1	casein kinase 1
CM	conditioned medium
CML	carboxymethyllysine
EDTA	ethylenediaminetetraacetic acid
FBS	fetal bovine serum
GAPDH	glycerinaldehyde 3-phosphate dehydrogenase
GO	glyoxal
GSK3β	glycogen synthase kinase-3β
IB	immunoblot
IP	immunoprecipitation
LEF-1	lymphoid enhancer-binding factor-1
LRP	low-density lipoprotein-related protein
MGO	methylglyoxal
NGF	nerve growth factor
PBS	phosphate buffered saline
PCR	polymerase chain reaction
PDGF	platelet-derived growth factor
PI3K	phosphoinositide 3-kinase
RAGE	receptor of advanced glycation end products
ROS	reactive oxygen species
RT-PCR	reverse transcription-PCR
TCF-4	T-cell factor 4
